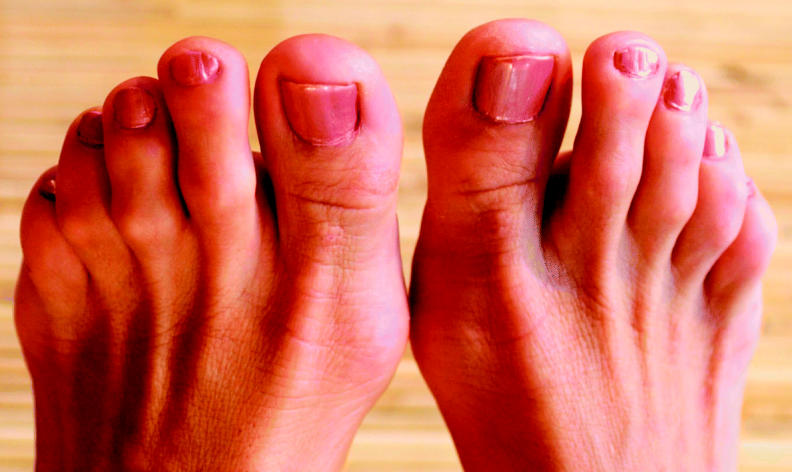# Headliners: Skin Cancer: Linking Toenail Arsenic Content to Cutaneous Melanoma

**Published:** 2005-06

**Authors:** Jerry Phelps

Beane Freeman LE, Dennis LK, Lynch CF, Thorne PS, Just CL. 2004. Toenail arsenic content and cutaneous melanoma in Iowa. Am J Epidemiol 160:679–687.

Although exposure to arsenic has been associated with increased risk of non-melanoma skin cancer, little research has been conducted on possible links between arsenic and cutaneous melanoma. Now NIEHS grantees Laura E. Beane Freeman of the National Cancer Institute and the University of Iowa and Peter Thorne of the University of Iowa have joined with their colleagues to perform what may be the first epidemiologic study to investigate the association between cutaneous melanoma and environmental arsenic exposure.

Cutaneous malignant melanoma incidence is increasing in the United States, and its annual percentage increase is one of the highest for all cancers. This deadly cancer also has the lowest survival rate of all skin cancers. In 2003, approximately 54,200 cases were diagnosed and 7,600 deaths were attributed to cutaneous melanoma in the United States.

The study was conducted in Iowa, where some areas have high drinking water concentrations of arsenic. The researchers identified participants through the population-based Iowa Cancer Registry. They selected 368 white Iowans aged 40 and older who had been diagnosed with cutaneous melanoma and 373 controls who had been diagnosed with colorectal cancer. Colorectal cancer was chosen as a control because it is a common cancer with no known link to arsenic. Study participants completed a survey and submitted toenail clippings for arsenic analysis.

The researchers found about a twofold increased risk of melanoma for participants with elevated toenail arsenic concentrations. Risk of melanoma with increasing toenail arsenic content was almost seven times greater for those reporting an earlier skin cancer diagnosis. Participants with the highest toenail arsenic levels were more likely to use private wells as compared to those with the lowest arsenic levels. Private well use is a known risk factor for arsenic exposure because wells are not held to the same testing requirements as public water supplies.

The observed higher correlation in people with a prior skin cancer diagnosis lends further support to a causal association between arsenic and cutaneous melanoma. The authors speculate that this finding may not have been previously reported because similar studies have been conducted primarily in Asian populations, who have a much lower risk of melanoma than Caucasians. The authors further suggest that genetic factors such as skin color may modify the effect of arsenic on melanoma risk. Further research must be conducted to confirm these results and clarify the link between arsenic exposure and development of melanoma.

## Figures and Tables

**Figure:**